# One-Dimensional P-Doped Graphitic Carbon Nitride Tube: Facile Synthesis, Effect of Doping Concentration, and Enhanced Mechanism for Photocatalytic Hydrogen Evolution

**DOI:** 10.3390/nano12101759

**Published:** 2022-05-21

**Authors:** Dazhuang Yu, Tiekun Jia, Zhao Deng, Qichen Wei, Kun Wang, Lihua Chen, Pingping Wang, Jiedong Cui

**Affiliations:** 1 State Key Laboratory of Advanced Technology for Materials Synthesis and Processing, Wuhan University of Technology, Wuhan 430070, China; ydz@whut.edu.cn (D.Y.); 290955@whut.edu.cn (Q.W.); Kkkun@whut.edu.cn (K.W.); chenlihua@whut.edu.cn (L.C.); 2 School of Materials Science and Engineering, Henan Province International Joint Laboratory of Materials for Solar Energy Conversion and Lithium Sodium Based Battery, Luoyang Institute of Science and Technology, Luoyang 471023, China; 3 State Key Laboratory of Advanced Technology for Float Glass, CNBM Research Institute for Advanced Glass Materials Group Co., Ltd., Bengbu 233000, China; wangpingping@ctiec.net (P.W.); cuijiedong@ctiec.net (J.C.); 4 Silica-Based Materials Laboratory of Anhui Province, Bengbu 233000, China

**Keywords:** P-doped, g-C_3_N_4_, porous wall, surface defect, photocatalytic hydrogen evolution

## Abstract

P-doped graphitic carbon nitride tubes (P-CNTS) with different P concentrations were successfully fabricated via a pre-hydrothermal in combination with a calcination process under a nitrogen atmosphere. The as-prepared samples exhibited excellent photocatalytic performance with a hydrogen production rate (HPR) of 2749.3 μmol g^−1^ h^−1^, which was 17.5 and 6.6 times higher than that of the bulk graphitic carbon nitride (CNB) and graphitic carbon nitride tube (CNT). The structural and textural properties of the P-CNT samples were well-investigated via a series of characterization methods. Compared with the bulk g-C_3_N_4_, the tubular structure of the doped samples was provided with a larger specific surface area (SSA) and a relatively rough interior. Besides the above, surface defects were formed due to the doping, which could act as more active sites for the hydrogen production reaction. In addition, the introduction of the P element could effectively adjust the band-gap, strengthen the harvest of visible-light, and boost the effective separation of photogenerated charges. More interestingly, these findings can open up a novel prospect for the enhancement of the photocatalytic performance of the modified g-C_3_N_4_.

## 1. Introduction

Nowadays, we are facing a global energy crisis caused by a growing population, increased demand, and excessive energy consumption. Therefore, it is urgent to explore efficient and sustainable technologies to generate clean and renewable energy. Recently, semiconductor photocatalytic hydrogen production has received extensive attention, which can effectively alleviate the serious energy crisis [1,2,3].

Recently, different semiconductor materials (e.g., TiO_2_ [4], ZnO [5], CdS [6], Bi_2_WO_6_ [7], CaTiO_3_ [8], ZnIn_2_S_4_ [9], g-C_3_N_4_ [10,11,12,13,14] etc.) have been reported for photocatalytic hydrogen production. Among these semiconductors, g-C_3_N_4_ (CNB) has been widely studied because of its low cost, earth-abundant, non-toxic, stable physicochemical properties, and suitable energy band structure [10,11,15]. However, the CNB sample has the disadvantages of low visible-light utilization and high-photogenerated carrier (electron–hole pairs) quenching rate, which have greatly limited its application in photocatalytic hydrogen production [10,11,12,13,14]. Many strategies have been developed to address these problems such as morphology modulation [16,17], the integration with carbon nanodots [18], and the construction of a heterogeneous structure with other semiconductors (NiO [19], BiOI [20], BiOCl [21], Cu_2_O [22], MoS_2_ [23] etc.). However, this modification has still failed to significantly improve the hydrogen production activity of CNB. Compared with the modification of constructing a hetero-structure and element doping (B [24], I [25], O [1,14,26], C [27], Fe [28], P [11,29,30,31,32] etc.) are considered as the most effective strategies to adjust the band-gap and decrease the quenching rate of the photogenerated electron–hole pairs, which led to the improvement in the photocatalytic performance. For example, Zhou et al. successfully synthesized the P-doped g-C_3_N_4_ by thermally induced copolymerization [11]. When the design amount of the P element was 2.5 wt% of the precursor, the as-prepared sample had the best hydrogen production performance, where the HPR could reach 506 μmol g^−1^ h^−1^, which was 2.9 times higher than that of the bulk g-C_3_N_4_. The improved performance can be attributed to the introduction of the P element, which decreased the band-gap, enhanced the visible-light response range, and hindered the recombination of the photogenerated charges. Ran et al. prepared P-doped graphitic carbon nitride nanosheets (PCN-S) for the first time by combining the P-doped and thermal exfoliation methods [30]. The prepared sample had the highest apparent quantum efficiency of 3.56% at 420 nm when the P element was fed at 0.5 wt% of the precursor, which was the highest of the g-C_3_N_4_ nanosheet materials. Therefore, the PCN-S sample possessed the highest photocatalytic HPR (1596 μmol g^−1^ h^−1^), which was 14.8 times higher than the bulk g-C_3_N_4_ (108 μmol g^−1^ h^−1^). Guo et al. obtained P-doped tubular g-C_3_N_4_ (P-TCN) and the photocatalytic HPR was 570 μmol g^−1^ h^−1^, which was 9.5 times higher than the pristine g-C_3_N_4_ [32]. The content of the P element in the sample with the best hydrogen production performance was explored to be 0.87 wt% when further analyzed by XPS. The experimental results showed that the introduction of the P element could effectively improve the absorption of visible-light, boost the separation and migration of the photoexcited carriers, and enhance the performance of g-C_3_N_4_.

Although many works on the P-doped and morphology modulation have been reported, they only fixed the P concentration and failed to delve into the connection between the doping concentration and the photocatalytic performance [32]. In addition, Putri et al. fabricated O-doped g-C_3_N_4_ (O-gC_3_N_4_x) with different O-doped concentrations by the hydrothermal method [33], where x = 10, 30, 50, 70, 90, and the O concentration was controlled via the addition of hydrogen peroxide. The experimental results showed that O-gC_3_N_4_30 possessed the optimal catalytic performance and the hydrogen production performance of the remaining O-doped g-C_3_N_4_ was inferior to that of O-gC_3_N_4_30. This demonstration confirmed the importance of the doping level of the impurity elements, which can greatly affect the hydrogen production performance of the photocatalyst. Based on the above, we intended to vary the P source dosage and investigate the effect of different doping concentrations on the photocatalytic performance of the samples and explore the mechanism of the enhanced photocatalytic performance in depth. In our work, the P-CNT samples with different P contents were synthesized, and the photocatalytic property was evaluated by decomposing aquatic hydrogen. As expected, the as-obtained samples had the optimal photocatalytic performance when the content of P element was 0.9 wt%, with a photocatalytic HPR of 2749.3 μmol g^−1^ h^−1^, which was 17.5 and 6.6 times higher than the CNB and CNT samples, respectively. In addition, we also propose a reasonable mechanism to explain the enhanced performance.

## 2. Materials and Methods

### 2.1. Materials

Melamine (C_3_H_6_N_6_), triethanolamine (TEOA), and chloroplatinic acid hexahydrate (H_2_PtCl_6_·6H_2_O) were all obtained from Aladdin Chemical Reagent Co., Ltd. (Shanghai, China). Disodium hydrogen phosphate dodecahydrate (Na_2_HPO_4_·12H_2_O) was obtained from Sinopharm Chemical Reagent Co., Ltd. (Shanghai, China).

### 2.2. Synthesis of Photocatalyst

**Scheme 1:**
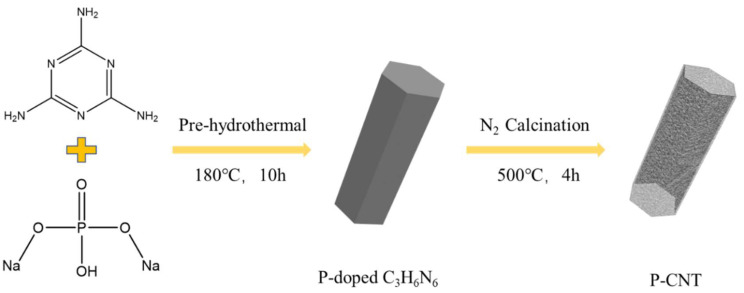
Schematic illustration for the synthesis of P-CNT.

CNT and P-CNT with different P contents were synthesized via a pre-hydrothermal in combination with the calcination process under a nitrogen atmosphere. The specific preparation flow chart of P-CNT is concisely depicted in Scheme 1. In brief, 1.26 g of melamine was dissolved in 100 mL of deionized water under a water bath at 85 °C, and then different masses of disodium hydrogen phosphate dodecahydrate (Na_2_HPO_4_·12H_2_O) were added to the solution and vigorously stirred for half an hour. Subsequently, the solution was then transferred to the polytetrafluoroethylene liner, then the reactor was screwed down and held at 180 °C for 10 h. After the autoclave was cooled naturally, the mixed solution was centrifuged, washed with deionized water and anhydrous ethanol, and dried in an oven at 60 °C. Finally, the as-obtained solid powder was heated to 500 °C with a heating rate of 2.5 °C/min and held for 4 h under a nitrogen atmosphere. Additionally, the concentrations of P element were designed to be 0.3 wt%, 0.6 wt%, 0.9 wt%, 1.2 wt% and 1.5 wt%, and the corresponding samples were marked as 0.3P-CNT, 0.6P-CNT, 0.9P-CNT, 1.2P-CNT, and 1.5P-CNT, respectively. For comparison, the CNT photocatalyst was obtained in the absence of Na_2_HPO_4_·12H_2_O via a similar procedure. The CNB sample was acquired through the common air pyrolysis method.

### 2.3. Characterization

The XRD patterns were gained via a Bruker D8. The SEM images were acquired using a Hitachi S-4800 instrument operating at 5 kV. The TEM photographs were gained from JEOL JEM 1400F (JEOL Ltd., Tokyo, Japan) with an accelerating voltage of 120 kV, and high-angle annular dark-field scanning transmission electron microscopy (HAADF-STEM) photographs were acquired from FEI Talos F200S (Thermo Fisher Scientific, New York, NY, USA). The SSA of samples was determined using a Tristar II 3020 surface area and porosity analyzer (Micromeritics, Norcross, GA, USA) according to the Brunauer–Emmett–Teller (BET) method. The UV–Vis diffuse reflectance spectrum was obtained by a PuXi TU-1901 UV–Vis spectrophotometer (Puxi, Beijing, China), with BaSO_4_ as the reference. XPS patterns were tested using a Thermo Fisher Scientific ESCALAB 250Xi (Thermo Fisher Scientific, New York, NY, USA) with a monochromatic Al-Kα line source, where the standard C1s peak centered at 284.8 eV was used as the reference. The electron paramagnetic resonance (EPR) patterns were obtained via a Bruker EMX-nano (Bruker, Karlsruhe, Germany).

### 2.4. Photocatalytic Hydrogen Production Test

The photocatalytic hydrogen production experiment was conducted in a Pyrex top-irradiated reactor connected to a sealed glass gas-circulation system. The light source was a 300 W Xenon lamp (PLS-SEX300C, Perfectlight Technology Co., Ltd., Beijing, China) with a 420 nm cut-off filter. A total of 50 mg of photocatalyst was homogeneously dispersed in a mixture of 80 mL of water and 20 mL of triethanolamine by sonication and stirring. In addition, 500 μL of 1 mg/mL H_2_PtCl_6_ solution (1 wt%) was in situ photo-deposited onto the sample surface as a cocatalyst. The reactant system was evacuated to eliminate air from the vessel prior to the light testing. During the photocatalytic reaction, the temperature of the circulating condensate was retained at 5 °C. The generated gases were analyzed online via gas chromatography (GC7900, Agilent, Santa Clara, CA, USA) equipped with a 5Å molecular sieve filled column and a thermal conductive detector (TCD), using high purity nitrogen as the carrier gas.

### 2.5. Photoelectrochemical Measurement

The photoelectrochemical properties of all samples were tested via the electrochemical workstation connected to a three-electrode system equipped with a counter electrode (Pt plate), a reference electrode (Ag/AgCl), and a working electrode (an FTO glass plate loaded photocatalyst). A neutral Na_2_SO_4_ solution (0.5 M, pH = 6.8) was selected as the electrolyte to conduct the charge.

Furthermore, the working electrode was manufactured as follows: First, a mixture consisting of 3 mg photocatalyst, 250 μL deionized water, 250 μL anhydrous ethanol, and 10 μL DuPont D1020 Nafion solutions (5 wt%) was uniformly dispersed with ultrasound for 20 min. Then, 25 μL of the as-prepared mixture was uniformly dropped on an FTO glass with the size 1 × 1 cm^2^. Finally, the FTO glass was dried under the irradiation of an infrared lamp.

## 3. Results and Discussion

**Figure 1:**
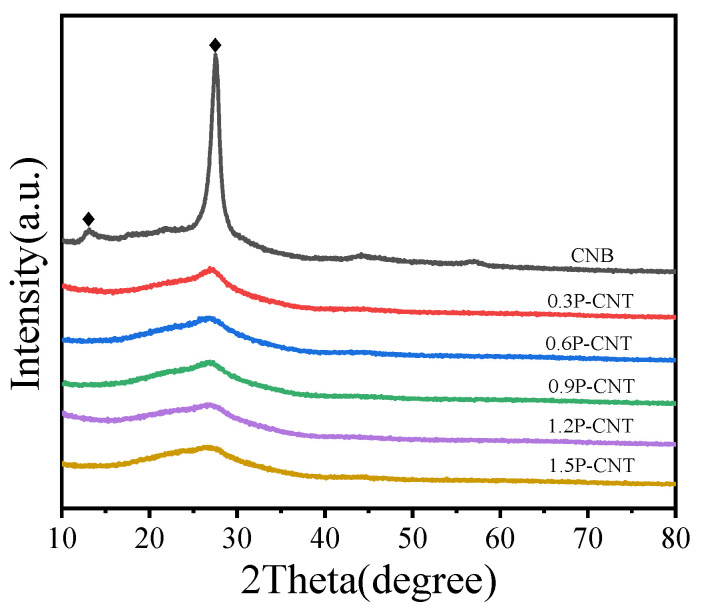
The XRD patterns of the CNB, 0.3P-CNT, 0.6P-CNT, 0.9P-CNT, 1.2P-CNT, and 1.5P-CNT samples.

The X-ray diffraction patterns were investigated to explore the influence of P-doped on the crystal structure of the CNB. As marked in Figure 1, the CNB sample possessed two prominent and sharp diffraction peaks at around 13.2° and 27.7°, which derived from the in-plane structural stacking (100) and cyclical stacking (002) of layers following the c-axis direction [10,34,35], respectively. However, it was noted that the diffraction peak at 13.2° for all the P-CNT samples disappeared and the diffraction peak at 27.7° also became wider and weaker than that of the CNB, mainly due to the hollow structure in the plane and the introduction of the P element, indicating that the P element possesses the effect of reducing the interlayer periodicity due to the mismatch of crystal parameters.

**Figure 2:**
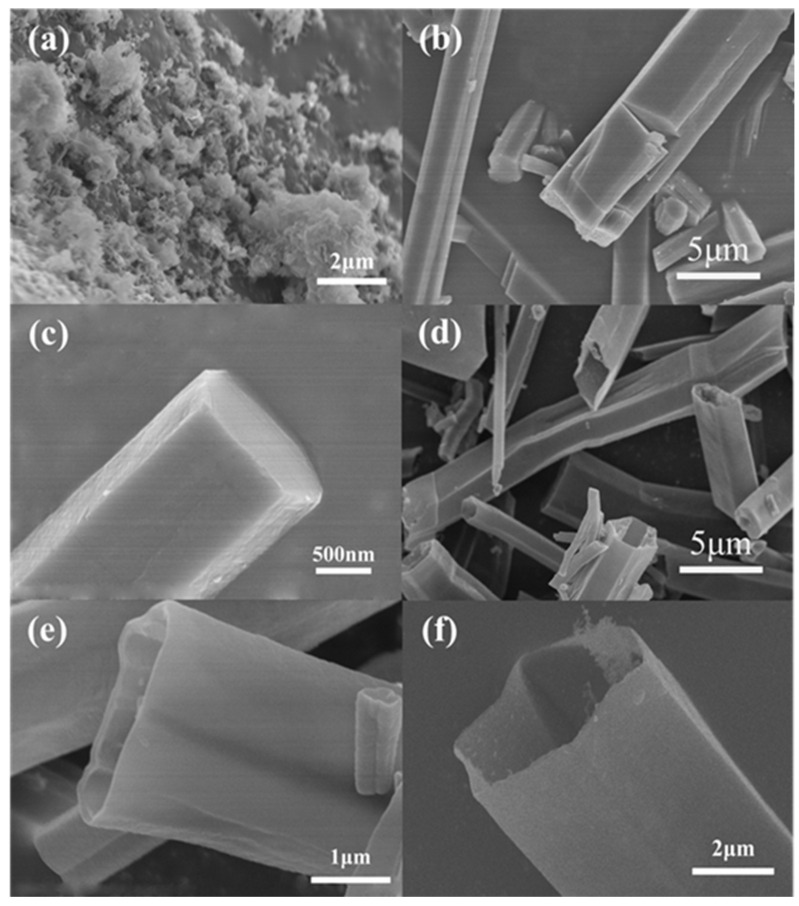
The SEM images of the CNB (**a**), low magnification (**b**), and high magnification (**c**) of the supramolecular precursors, and low magnification (**d**) and high magnification (**e**,**f**) of the 0.9P-CNT sample.

The morphology and microstructure of the CNB and 0.9P-CNT samples were observed via SEM and TEM images. As displayed in Figure 2a, the CNB sample exhibited a typical blocky structure with an irregular and heterogeneous arrangement [36]. After hydrothermal treatment, a supramolecular precursor with a rod-like structure could be obtained (Figure 2b,c), originating from the in situ self-assembly process of melamine and its hydrolyzed cyanuric acid [32]. The rod-shaped precursors were of different sizes and randomly intercrossed. The morphology of the 0.9P-CNT sample is shown in Figure 2d–f, where we can see that it possessed a tubular structure with a relatively rough surface and hollow shape with a diameter of about 1–5 μm. When the rod precursor was calcined, the decomposition may have started from the inside first, and the groups containing the C and N elements in the sample were decomposed and released as gases. Then, the carbon nitride with a tubular structure could be formed under the impact of the flowing N_2_ atmosphere. The sizes of the as-obtained tubular samples were inhomogeneous due to the inhomogeneous sizes of the precursors.

**Figure 3:**
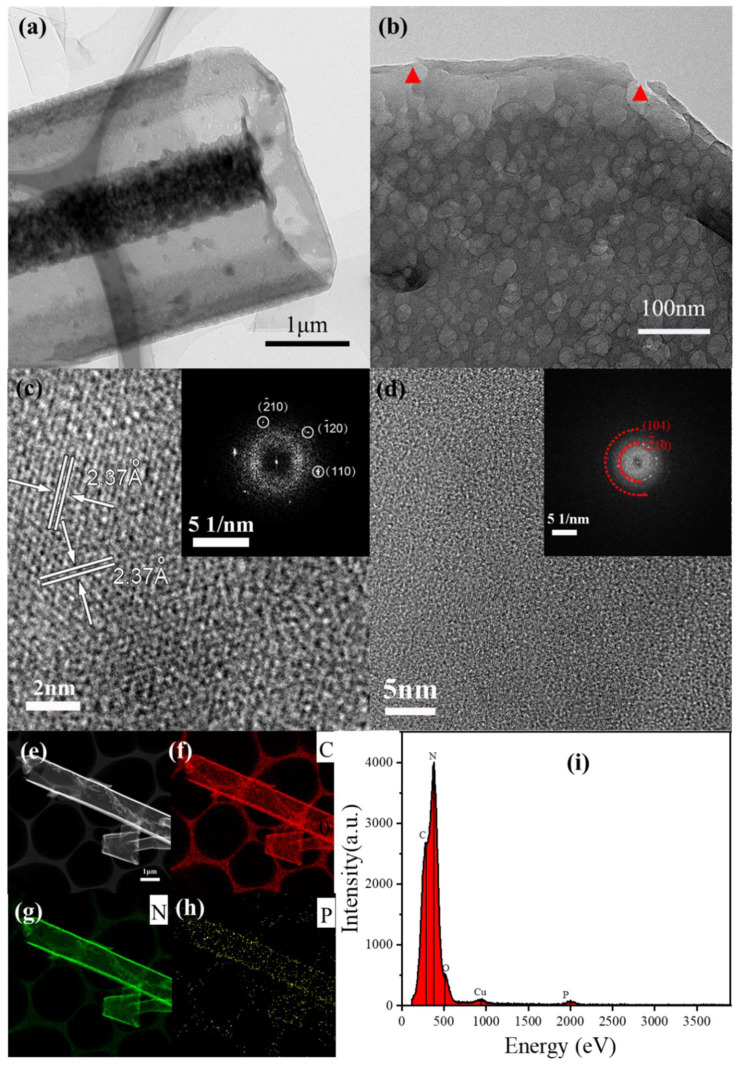
The TEM images (**a**,**b**) and HRTEM images (**c**,**d**) of the 0.9P-CNT sample, the inset is the corresponding selected area electron diffraction image, HAADF-STEM image and element mapping images of C, N, and P for the 0.9P-CNT sample (**e**–**h**), EDX spectrum of the 0.9P-CNT sample (**i**).

**Figure 4:**
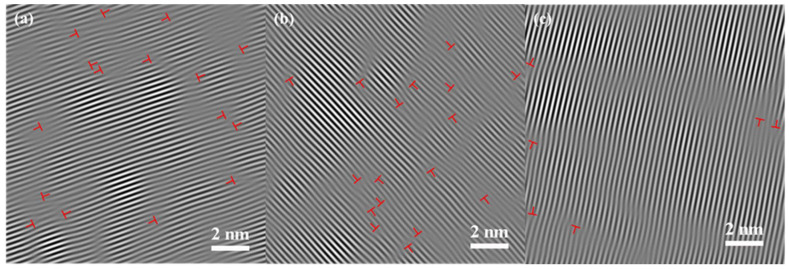
(**a**) ($\bar{2}10$), (**b**) ($\bar{1}20$), (**c**) (110) inverse Fourier transform images in the single crystal region.

In addition, Figure 3a shows the TEM image of the 0.9P-CNT sample, which also demonstrates that 0.9P-CNT has a tubular structure with a diameter around 1–3 μm. In addition, the interior of the tubular structure is rough, which can provide more active sites [37,38,39] and promote more efficient charge transfer, further enhancing the photocatalytic performance. More importantly, as seen in Figure 3b, there were a large number of pores in the walls of the tubular structure with a diameter of 30–50 nm, resulting in a special porous structure, which can accelerate the release rate of hydrogen during the hydrogen production process [17,40,41]. Figure 3c,d shows the high-resolution transmission electron microscopy (HRTEM) photographs of the 0.9P-CNT sample, and the inset shows the corresponding selected area electron diffraction images. From the few numbers of lattice stripes in the figure, we can see that the crystallinity of the as-prepared sample was very poor, corresponding to the XRD analysis, while Figure 3d confirms its polycrystalline nature. Figure 3e shows the HAADF-STEM photo of the 0.9P-CNT sample, and Figure 3f–h are the corresponding element mapping images. As illustrated in Figure 3f,g, the C and N elements were uniformly distributed in the tubular sample. Furthermore, Figure 3h confirms that the P element was indeed present in the 0.9P-CNT sample, and the EDX analysis results in Figure 3i provide strong evidence for the doping of the P element into the CNT. More interestingly, inverse Fourier transform images of different orientations could clearly be seen in Figure 4, suggesting crystal distortions in the 0.9P-CNT sample. Specifically, the red symbol T in the figure indicates the defects present in the sample [42], and many symbols of T revealed that the as-prepared sample possessed a large number of surface defects.

**Figure 5:**
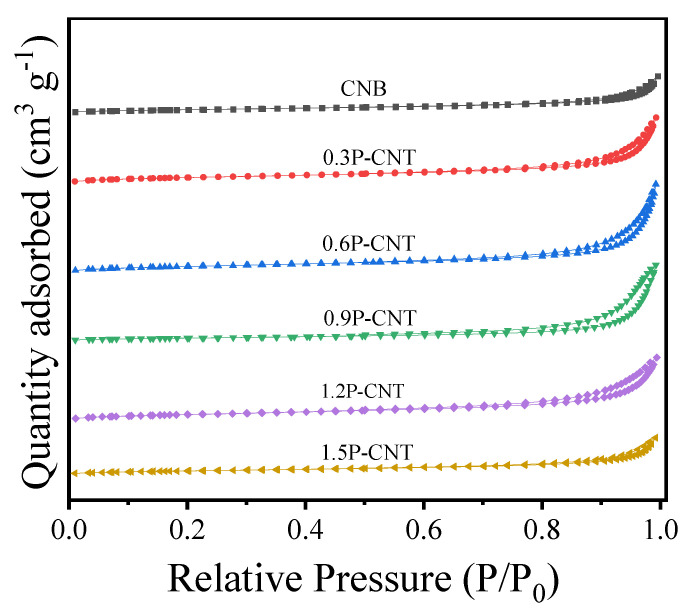
N_2_ adsorption-desorption curves of the CNB, 0.3P-CNT, 0.6P-CNT, 0.9P-CNT, 1.2P-CNT, and 1.5P-CNT samples.

The N_2_ adsorption–desorption curves of the CNB and P-CNT samples are displayed in Figure 5, where all samples exhibited the type IV isotherm, indicating that the as-prepared samples possessed well-defined mesopores from the tubular structure and porous walls [32], which agreed well with the analytical results of the SEM and TEM. Table 1 shows the SSA values of the CNB and P-CNT samples, from which it can be seen that 0.9P-CNT had the highest SSA value. As a consequence, the increased SSA and the amorphous characteristic indicated that more active sites can be generated for the hydrogen production reaction [11,30], resulting in the improvement in the hydrogen production performance.

**Figure 6:**
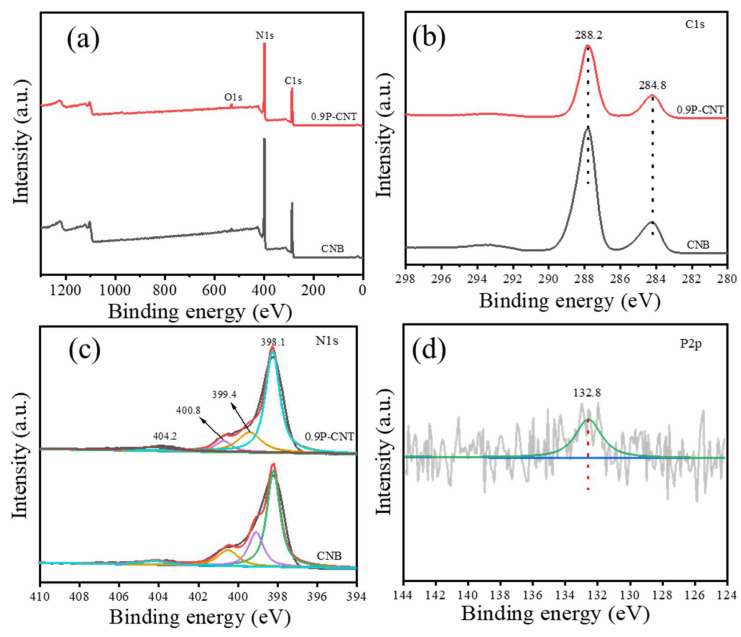
The XPS survey spectra of the as-prepared CNB and 0.9P-CNT samples (**a**), and high-resolution XPS spectra of CNB and 0.9P-CNT in different regions. (**b**) C1s, (**c**) N1s, and (**d**) P2p.

X-ray photoelectric spectroscopy (XPS) was employed to further investigate the surface elemental composition and chemical status of the P bonded with the other elements. Figure 6a demonstrates the XPS survey spectra, in which peaks belonging to the C, N, and O elements could be observed in the XPS survey spectrum of the 0.9P-CNT sample. The signal of the O element originated from the oxygen adsorbed in the air, however, the absence of the P element detected in the survey spectrum may be due to its low content. To further investigate the interaction of the P element with CNT, the high-resolution XPS tests were performed and the results are displayed in Figure 6b–d. In the C 1s spectrum (Figure 6b) of the 0.9P-CNT sample, two significantly dominant peaks could be found at 284.8 and 288.2 eV, corresponding to the adventitious carbon and the sp^2^-hybridized carbon containing defects (-N=C-N), respectively [43,44,45]. The N 1s spectrum (Figure 6c) of the 0.9P-CNT sample can be subdivided into four smaller peaks with binding energies of 398.1, 399.4, 400.8, and 404.2 eV [46,47]. The major N 1s peak at 398.1 eV originated from the sp^2^-hybridized nitrogen atom in the triazine rings (C=N-C), while the peak at 399.4 eV was associated with the N-(C)_3_ group or the amino group ((C)_2_-N-H) in the sp^3^-hybridization. A weak peak at 400.8 eV corresponded to the N-(C)_3_ groups or a residual amino functional group in the aromatic cycle. In addition, there was an ignorable peak at 404.2 eV from the π excitation [47,48]. The feeble peak at 132.8 eV in Figure 6d was derived from the P–N bond [31,32], further indicating the presence of the P element in the 0.9P-CNT sample. On this basis, the XPS analysis results also strongly demonstrate that P successfully substituted C and formed the P–N bond in the 0.9P-CNT sample, leading to the formation of crystal defects, which would exert a positive effect in separating the photogenerated electron–hole pairs.

**Figure 7:**
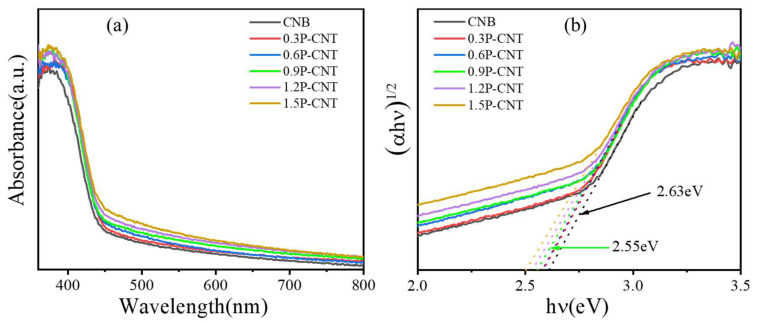
The UV–Vis diffuse reflectance spectra (**a**) and corresponding Tauc plot (**b**) of the CNB, 0.3P-CNT, 0.6P-CNT, 0.9P-CNT, 1.2P-CNT, and 1.5P-CNT samples.

It is well-acknowledged that the light absorption property of a semiconductor is intimately correlated with the energy band structure. Therefore, different photocatalysts exhibit different a light absorption property in the visible-light absorption range. Consequently, the diffuse reflectance spectra (DRS) of all samples were measured, and the results are displayed in Figure 7a. As demonstrated in the figure, the CNB sample possessed a distinct absorption edge at about 470 nm. By introducing the P element into the CNB sample, all P-CNT samples exhibited improved visible-light absorption in the visible region due to the substitution of P atoms for C to form P–N bonds [31,32]. The above results verify that the introduction of the P element can improve the light absorption of CNB, further generating more photoexcited electron–hole pairs involved in the photocatalytic reaction. In fact, the capture of visible-light could also be enhanced due to the multiple reflection effect of the incident light inside the 0.9P-CNT sample. The corresponding band-gap values from the Tauc plot (Figure 7b) decreased from 2.63 eV (CNB) to 2.55 eV (0.9P-CNT), which also indicated that such a modification strengthened the visible-light absorption. As previously reported [49], doping P atoms into g-C_3_N_4_ instead of C shifted the VBM and CBM down by 0.22 eV and 0.73 eV, respectively. These changes are consistent with previous reports that hetero-element-doped conjugated polymers have a tendency to reduce the band-gap width [50,51,52].

**Figure 8:**
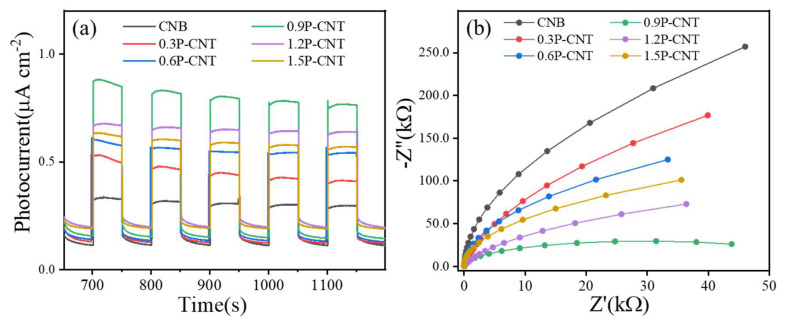
The photocurrent (**a**) and electrochemical impedance spectra (**b**) of the CNB, 0.3P-CNT, 0.6P-CNT, 0.9P-CNT, 1.2P-CNT, and 1.5P-CNT samples.

According to previous reports in the literature [10,35], the photocurrent response of semiconductors can effectively reflect the separation and migration of photogenerated carriers. Thus, photocurrent tests of the as-prepared samples were performed, and the results are demonstrated in Figure 8a. It was found that the photocurrents of all P-CNT samples were larger than that of CNB sample, suggesting that the introduction of the P element could accelerate the separation of photogenerated carriers and hinder the complexation between them, thus allowing more photogenerated electrons to participate in the reduction reaction. Among them, the 0.9P-CNT sample possessed the highest photocurrent response, indicating that the most efficient separation and transport of the photogenerated charges were obtained in the 0.9P-CNT sample. In addition, the separation efficiency of the interfacial charges was further explored via electrochemical impedance spectroscopy (EIS). According to previous reports [34,53], the smaller the radius of the circular arc, the lower the resistance to interfacial charge transfer from the catalyst to the reaction molecule, and the easier the separation and migration of the photogenerated charges. The EIS Nyquist plots of the CNB and all of the P-CNT samples are demonstrated in Figure 8b. Obviously, it can be seen in the figure that the 0.9P-CNT sample had the smallest radius of a circle, indicating that the as-prepared 0.9P-CNT material had the smallest internal resistance and so had more effective separation of its photogenerated charges. The results of the EIS together with the photocurrent. support that the 0.9P-CNT sample possessed the most efficient charge separation, and therefore the optimal photocatalytic hydrogen production performance can be obtained.

**Figure 9:**
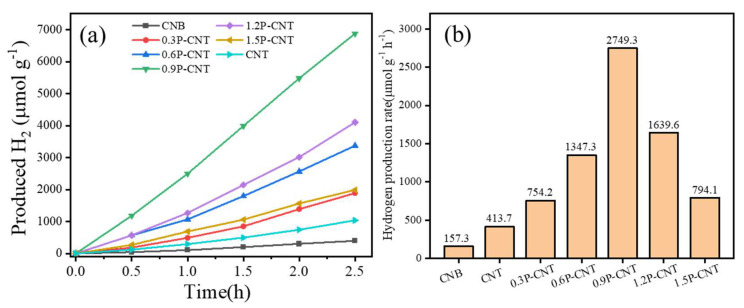
The photocatalytic hydrogen production (**a**) and HPR (**b**) of the CNB, 0.3P-CNT, 0.6P-CNT, 0.9P-CNT, 1.2P-CNT, 1.5P-CNT, and CNT samples.

An energy band structure suitable for photocatalytic hydrogen production makes the P modified materials promising visible photocatalysts. The photocatalytic property was assessed by detecting the produced hydrogen under the illumination of a Xenon lamp source (λ > 420 nm). Figure 9a shows the photocatalytic hydrogen production performance of the samples, from which it could be observed that the hydrogen production of the CNB and CNT was very low. The HPR of the CNB sample was only 157.3 μmol g^−1^ h^−^^1^, which stemmed from its narrow visible-light response range and fast-photogenerated carrier complexation rate [12,18]. The morphological modulation from the CNB to CNT (413.7 μmol g^−1^ h^−1^) increased the HPR, which illustrates that the tubular structure can improve the photocatalytic performance, but such a slight improvement is far from the desired goal. Excitingly, the HPR of all of the P-doped samples was higher than the CNB sample. With the increase in the P-source input, the HPR of P-CNT increased. When the P-source input reached 0.9 wt%, the 0.9P-CNT photocatalyst showed the highest HPR of 2749.3 μmol g^−1^ h^−1^, which was 17.5 and 6.6 times higher than the CNB and CNT samples, respectively. However, the HPR of the P-CNT gradually declined when the amount of P was further increased, indicating that too much element doping does not further enhance the catalytic performance, but causes negative effects. Furthermore, the 0.9P-CNT sample showed a better photocatalytic performance for the decomposition of aquatic hydrogen compared with most of the photocatalysts reported in the previous literature (Table 2).

**Figure 10:**
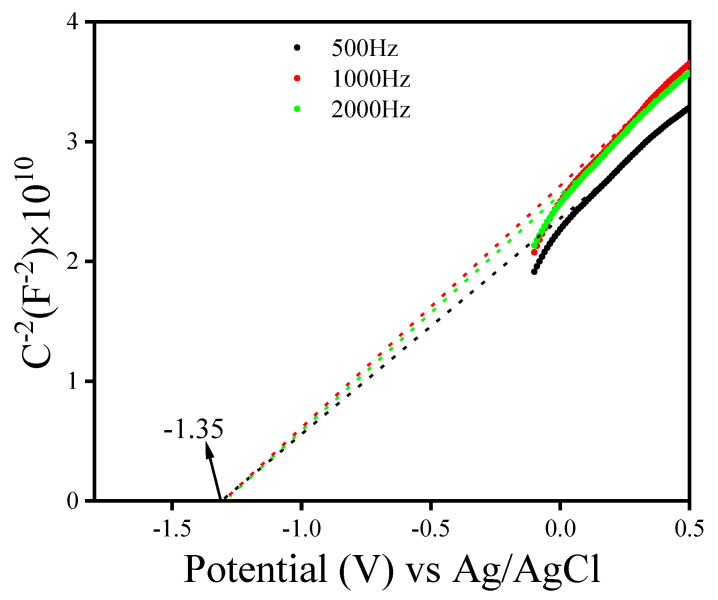
The Mott–Schottky plot of the 0.9P-CNT sample.

The energy band structure of the 0.9P-CNT sample was studied using the Mott–Schottky (M–S) plot to ensure the band edge potential and semiconductor nature. As shown in Figure 10, the M–S plot of the 0.9P-CNT sample exhibited a positive slope, indicating a typical n-type semiconductor [55]. In addition, the flat band (fb) potential of the 0.9P-CNT sample was estimated to be −1.35 V vs. Ag/AgCl. Subsequently, its E_fb_ (NHE) was determined using the Eq. E_fb_ (NHE) = E_fb_ (Ag/AgCl) + (0.059 × pH) + E(AgCl), where the pH of the Na_2_SO_4_ electrolyte was 6.8 and E(AgCl) was 0.198 V at 25 °C. Usually the E_CBM_ = E_fb_ (NHE) −0.2 V for n-type semiconductors, therefore, the E_CBM_ of 0.9P-CNT was evaluated to be −0.95 V. Based on the Eg value of the Tauc plot with E_CBM_, the E_VBM_ was estimated to be 1.6 V. Such a negative band can enable the 0.9P-CNT sample to achieve photocatalytic hydrogen production and become a promising hydrogen production photocatalyst.

**Figure 11:**
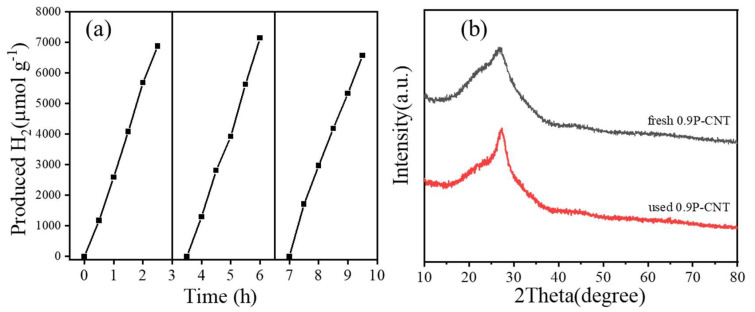
The cyclic hydrogen production test of the 0.9P-CNT sample (**a**) and the XRD patterns of the fresh and used 0.9P-CNT samples (**b**).

The performance stability is very significant for the commercial use of photocatalysts, so the 0.9P-CNT sample was used for the cyclic hydrogen production test under the same conditions and the experimental results are shown in Figure 11a. After three cycles of hydrogen production tests, the amount of produced hydrogen slightly decreased. In addition, the fresh and used 0.9P-CNT samples were characterized by XRD, and the results (Figure 11b) showed that the crystal structure of the 0.9P-CNT sample remained intact after the cyclic tests, indicating that the 0.9P-CNT photocatalyst possessed excellent photostability.

**Figure 12:**
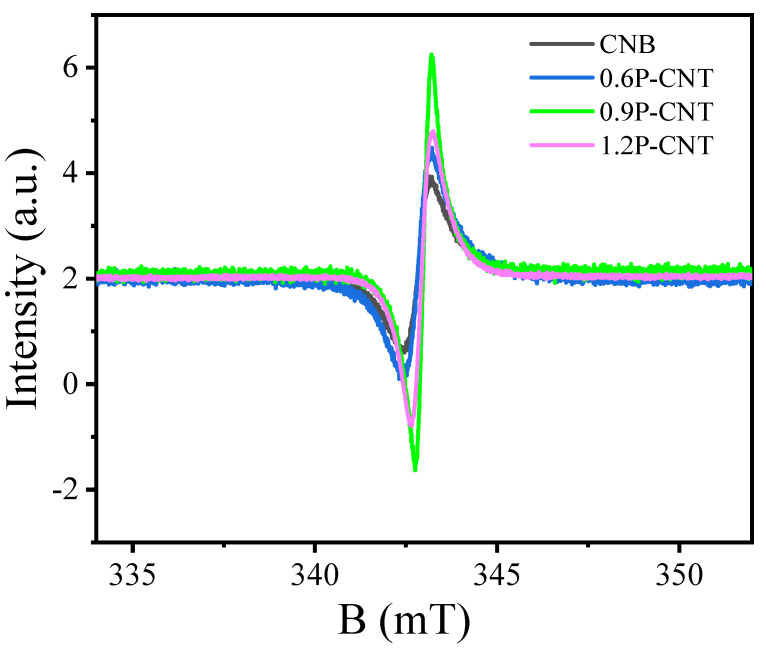
Room temperature EPR signals for the CNB, 0.6P-CNT, 0.9P-CNT, and 1.2P-CNT samples.

To analyze the generation of unpaired electrons in the catalyst, the prepared CNB, 0.6P-CNT, 0.9P-CNT, and 1.2P-CNT samples were measured by EPR spectroscopy at room temperature, and the results are shown in Figure 12. As can be seen in Figure 12, only one Lorentz line centered at the g value of 2.0034 was observed in the magnetic field from 346.5 to 365 mT for all samples. In a typical graphitic carbon nitride structure, the g value equal to 2.0034 was attributed to the lone pair of electrons in the sp^2^-carbon [25]. In addition, the EPR intensity of the 0.6P-CNT, 0.9P-CNT, and 1.2P-CNT samples were all greater than that of the CNB when P atoms were introduced into the CNB. In particular, the 0.9P-CNT sample possessed the highest signal intensity, which indicates the presence of more unpaired electrons for the hydrogen production reactions in the 0.9P-CNT sample. It is also possible to show that the P element can promote the gradual development of the electronic energy band structure and inhibit the compounding of photogenerated charges. Furthermore, the above result revealed that the doping concentration of P exerted a significant effect on the generation of unpaired electrons. The optimal value for the doping concentration was 0.9 wt%, and the largest generation of unpaired electrons was achieved, which agreed well with that of photocatalytic hydrogen evolution.

Based on the analysis of the above characterization results, we propose a rational mechanism to account for the enhanced hydrogen production performance. First, a porous tube structure with a larger SSA and rough inner surface could provide more active sites for the photocatalytic reaction. Simultaneously, the presence of a mesoporous structure could accelerate the release rate of hydrogen during the hydrogen production process. Second, the doping of P into CNB led to the formation of surface defects, which acted as active sites for the photocatalytic reaction. Third, the incorporation of the P element with the optimal concentration could effectively adjust the band-gap, strengthen the harvest of visible-light, and boost the effective separation of the photogenerated charges. However, it can be noted that an excess of incorporated P atoms would evolve into recombination centers of the photogenerated electron–hole pairs, thus reducing the hydrogen production performance. Thus, we can conclude that the remarkable improvement in the photocatalytic hydrogen evolution could be due to the synergistic effect of the augmentation of active sites and light absorption capacity as well as an enhanced separation rate of the photogenerated charges.

## 4. Conclusions

The hollow tubular 0.9P-CNT sample was obtained by the pre-hydrothermal treatment and nitrogen calcination of melamine, and the photocatalytic HPR of the 0.9P-CNT sample could reach 2749.3 μmol g^−1^ h^−1^, which was 17.5 and 6.6 times higher than that of the CNB and CNT samples, respectively. The enhanced photocatalytic performance can be attributed to the introduction of the P elements and a porous tubular structure. The porous tubular structure allowed the 0.9P-CNT sample to have a larger SSA and rougher inner surface, which can provide more active sites for the hydrogen production reaction, therefore, the introduction of the P element can lower the band-gap, enhance the light trapping rate, and promote the effective separation of the photogenerated charges. In summary, we believe that our study not only deepens the understanding of the enchantment mechanism of photocatalytic performance, but also offers the possibility of developing stable, efficient, and noble metal-free photocatalysts for practical applications.

## Figures and Tables

**Table 1:** The SSA of the CNB, 0.3P-CNT, 0.6P-CNT, 0.9P-CNT, 1.2P-CNT, and 1.5P-CNT.

Samples	SSA (m^2^ g^−1^)
CNB	9.4
0.3P-CNT	23.2
0.6P-CNT	25
0.9P-CNT	31.5
1.2P-CNT	26.3
1.5P-CNT	16.5

**Table 2:** The photocatalytic hydrogen production performance on the various reported photocatalysts.

Photocatalysts	Pt	HPR (μmol g^−1^ h^−1^)	Light Source (>420 nm)	References
porous crystalline g-C_3_N_4_	1 wt%	1010	LED (12 W)	[3]
CdS/P-g-C_3_N_4_ tube-1%	1 wt%	1579	300 W Xenon lamp	[15]
S-g-C_3_N_4_/g-C_3_N_4_ (0.3%)	1 wt%	359.6	LED (12 W)	[13]
g-C_3_N_4_	1 wt%	646	LED (12 W)	[2]
P-g-C_3_N_4_ tube	3 wt%	2020	300W Xenon lamp	[31]
P-TCN	1 wt%	570	300W Xenon lamp	[32]
P-g-C_3_N_4_-550	3 wt%	506	300 W Xenon lamp	[11]
P-g-C_3_N_4_ nanosheet	1 wt%	1596	300 W Xenon lamp	[30]
CNMS-600	3 wt%	3920	300 W Xenon lamp	[54]
0.9P-CNT	1 wt%	2749.3	300 W Xenon lamp	This work

## Data Availability

The data presented in this study are available in this article.
